# Potential Role of Ginger (*Zingiber officinale* Roscoe) in the Prevention of Neurodegenerative Diseases

**DOI:** 10.3389/fnut.2022.809621

**Published:** 2022-03-18

**Authors:** Raúl Arcusa, Débora Villaño, Javier Marhuenda, Miguel Cano, Begoña Cerdà, Pilar Zafrilla

**Affiliations:** Department of Pharmacy, Faculty of Health Sciences, Catholic University of San Antonio (UCAM), Murcia, Spain

**Keywords:** ginger, neurodegenerative diseases, Alzheimer's disease, Parkinson's disease, multiple sclerosis, gingerol, antioxidants

## Abstract

Ginger is composed of multiple bioactive compounds, including 6-gingerol, 6-shogaol, 10-gingerol, gingerdiones, gingerdiols, paradols, 6-dehydrogingerols, 5-acetoxy-6-gingerol, 3,5-diacetoxy-6-gingerdiol, and 12-gingerol, that contribute to its recognized biological activities. Among them, the major active compounds are 6-shogaol and 6-gingerol. Scientific evidence supports the beneficial properties of ginger, including antioxidant and anti-inflammatory capacities and in contrast, a specific and less studied bioactivity is the possible neuroprotective effect. The increase in life expectancy has raised the incidence of neurodegenerative diseases (NDs), which present common neuropathological features as increased oxidative stress, neuroinflammation and protein misfolding. The structure-activity relationships of ginger phytochemicals show that ginger can be a candidate to treat NDs by targeting different ligand sites. Its bioactive compounds may improve neurological symptoms and pathological conditions by modulating cell death or cell survival signaling molecules. The cognitive enhancing effects of ginger might be partly explained *via* alteration of both the monoamine and the cholinergic systems in various brain areas. Moreover, ginger decreases the production of inflammatory related factors. The aim of the present review is to summarize the effects of ginger in the prevention of major neurodegenerative diseases such as Alzheimer's disease, Parkinson's disease and multiple sclerosis.

## Introduction

The number of people over the age of 65 has progressively grown in Western countries, increasing the risk of age-related neurodegenerative diseases. The most common pathology is Alzheimer's disease, with more than 26 million people affected worldwide today. This number is expected to quadruple by 2050. No effective treatments are available for aging-related neurodegenerative diseases, which tend to progress in an irreversible manner and are associated with large personal and socioeconomic costs (1).

The prevention of these pathologies and the search for new nutraceuticals and drugs to combat them are the great challenges of scientific research. Plant-derived products are known to have protective effects including anti-inflammatory and antioxidant actions, related to improvements in cognitive impairment (2).

In recent years, several pharmacological activities of ginger and its bioactive compounds have been explored (3). *Zingiber officinale* is a perennial herb member of the *Zingiberaceae* family and its thick tuberous rhizomes is very popular for medicinal uses and as a spice and additive agent for flavoring foods and drinks (4, 5). Its origin is little known but it is thought to be in South-East Asia or India (4). The composition in bioactive compounds of *Zingiber officinale* varies according to the place where it is grown and the drying techniques. In general terms, the rhizome of *Zingiber officinale* is mainly composed of essential oils in small quantities, oleoresins, mineral salts, sugars, mucilage, starch, gums and organic acids. Starch constitutes 40–60% of the dry weight of the rhizome of *Zingiber officinale*. Ginger contains a variety of bioactive compounds responsible of its biological activities (as 6-gingerol, 6-shogaol, 10-gingerol, gingerdiones, gingerdiols, paradols, 6-dehydrogingerols, 12-gingerol 3,5-diacetoxy-6-gingerdioal and 5-acetoxy-6-gingerol), among which 6-gingerol and 6-shogaol stand out (6).

In recent years, ginger has been found to possess biological activities, such as antimicrobial, anti-inflammatory, antioxidant, anticancer (by improvement in the expression level of markers for colorectal cancer risk) and anti-allergic activities (7). In this sense, numerous studies have demonstrated that ginger possesses the potential to prevent cardiovascular diseases and associated pathologies that act as risk factors (diabetes, obesity and metabolic syndrome), chemotherapy-induced emesis and nausea, arthritis, gastric dysfunction, pain, respiratory disorders and neurodegenerative diseases (8, 9). Ginger could modulate obesity through various potential mechanisms including increasing lipolysis and thermogenesis, inhibition of lipogenesis, decrease of fat absorption and appetite control (10). Ginger has been documented to ameliorate hyperglycemia and hyperlipidemia. These beneficial effects are mediated by modulation of transcription factors, such as nuclear factor κB, peroxisome proliferator–activated receptors and adenosine monophosphate–activated protein kinase (11). In this sense, Zhu et al. (12) showed that ginger improves insulin sensitivity, decreases the levels of glycosylated hemoglobin in type 2 diabetes mellitus and ameliorates plasma lipid profile.

Neurodegenerative diseases are generally characterized by neuroinflammation, oxidative stress and protein misfolding than leads to brain damage, synaptic dysfunction and neuronal apoptosis (13). In Alzheimer's disease, oxidative stress is mainly caused by mitochondrial dysfunction, the intracellular accumulation of hyperphosphorylated tau (τ) proteins in the form of neurofibrillary tangles, the excessive accumulation of extracellular plaques of beta-amyloid (Aβ), as well as environmental and genetic factors. Gingerols have shown antioxidant, anti-amyloidogenic, anti-inflammatory and anti-cholinesterase properties (14). The major component extracted from *Zingiber officinale*, 6-gingerol, showed antioxidant and anti-inflammatory activity and inhibition of astrocyte overactivation. Lipopolysaccharide stimulated microglia induced pro-inflammatory cytokines, such as IL-6, IL-1β, increments of intercellular nitric oxide concentrations, as well as iNOS enzyme activity, and all of them were suppressed by the treatment with 6-gingerol (15).

Parkinson disease is the second most common neurodegenerative pathology after Alzheimer's disease (16). Its prevalence increases with age and is characterized by the accumulation of α-synuclein protein within neurons, inside Lewy neurites and Lewy bodies (17). Parkinson disease can be caused by environmental and hereditary factors, including iron accumulation in the brain and oxidative stress. Medeiros et al. (18) showed than oxidative stress levels and inflammatory markers were significantly increased in Parkinson disease patients. Mohd et al. (19) suggests that the active compound in ginger may reduce the associated cognitive dysfunction by inhibiting the inflammatory response, increasing levels of nerve growth factor and stimulating synapse formation.

Multiple sclerosis is characterized by chronic inflammatory response-induced demyelination of the neurons and degeneration of the axons within the central nervous system. Factors as inflammatory, oxidative and immunopathological parameters are related in the development and progression of multiple sclerosis. Ginger and its bioactive compounds could be considered as potential agents to treat multiple sclerosis due to their anti-inflammatory, antioxidant and immunomodulatory properties (20).

As oxidative stress and inflammation play an important role in the pathogenesis of the above mentioned diseases, the introduction of anti-inflammatory and antioxidant agents, such as ginger and derived products, could be useful for the treatment and prevention of neurodegenerative conditions. Hence, the aim of the present review is to summarize the effects of ginger in the prevention of major neurodegenerative diseases, focusing on Alzheimer's disease, Parkinson's disease and multiple sclerosis. For this purpose, literature search has been carried out consulting the scientific publications related to ginger published in Web of Science, Scopus, Science Direct and Pubmed databases. Articles published in the last 10 years have been selected, with some exceptions on publication date in those previous works of major relevance.

## Bioactive Compounds Present in Ginger

Rhizome of *Zingiber officinale* is composed of 69 volatile compounds, which constitute 97 % of its total composition in essential oils. Those molecules present at higher concentrations are α-Zingiberene (28,62%), Camphene (9,32%), Ar-curcumene (9,09%), β-Phellandrene (7,97%), E-α-Farnesene (5,52%), β-Bisabolene (5,40%), α-Pinene (2,57%) (21). It has been documented their biological properties such as antimicrobial, antioxidant, cytotoxic, insecticidal and anti-inflammatory effects as well as their usefulness to preserve food characteristics (22).

Non-volatile compounds (oleoresins) are the main source of bioactive compounds in the rhizome of *Zingiber officinale*. At present, 34 oleoresins have been discovered, which constitute 88.6% of the total composition (21), among which Gingerols (1-(4-hydroxy-3-methoxyphenyl)-5-hydroxyalcan-3-one), Shogaols (1-(4-hydroxy-3-methoxy-phenyl)-4-decen-3-one) and Paradols are the most important groups. Shogaols are the more abundant components in the dried rhizome and gingerols are mainly found in the fresh rhizomes of ginger (23).

**Figure 1:**
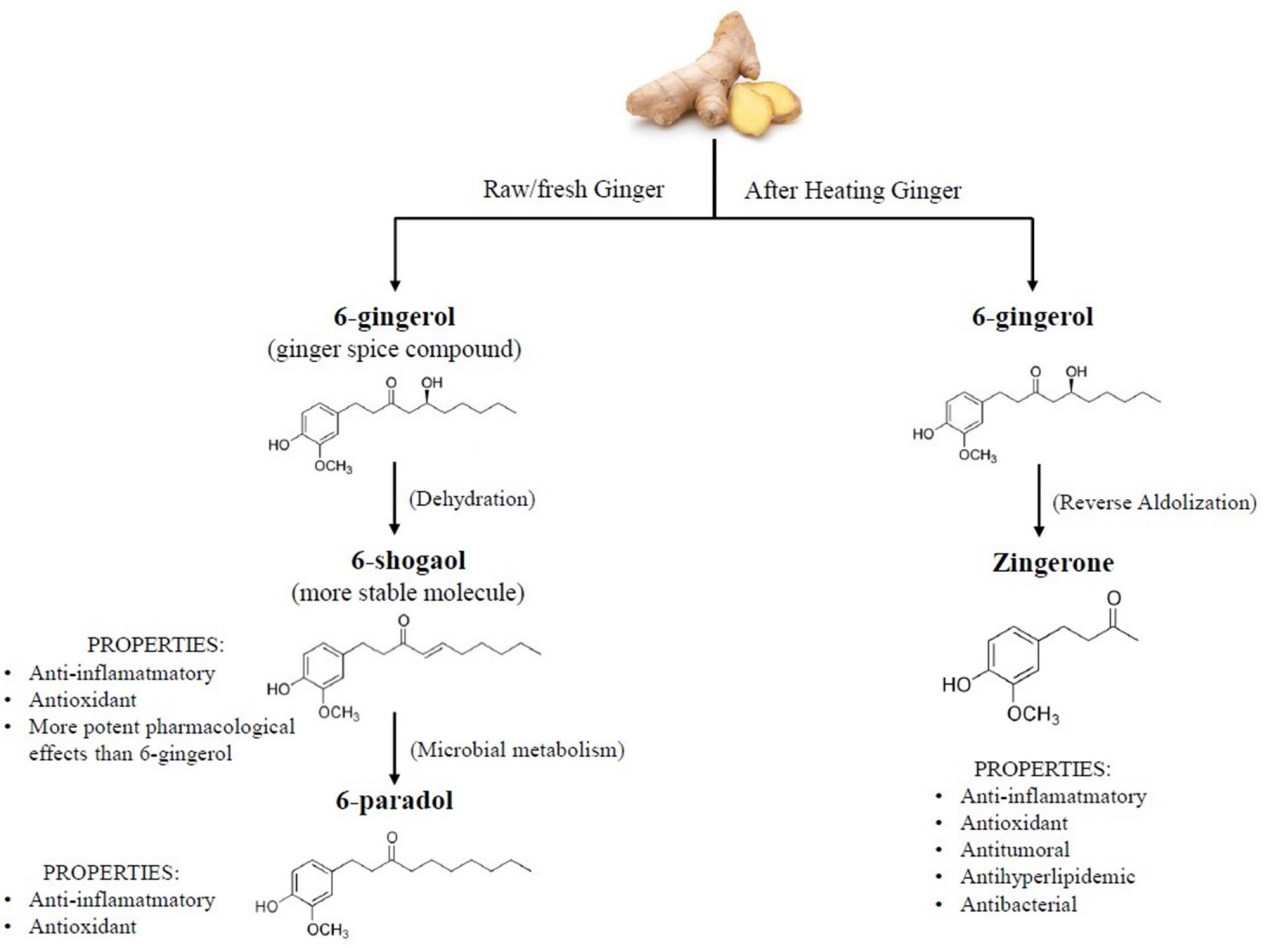
Chemical structures and properties of ginger bioactive compounds.

Gingerol analogs are thermally labile and undergo dehydration reactions to form the corresponding shogaols, which are more stable and have greater pharmacological effects than their precursors and are responsible of the characteristic pungent taste of dried ginger. This chemical change occurs in the process of thermal drying of the rhizomes and long-term storage (24). 6-shogaol is converted to 6-paradol by bacterial metabolism (13, 25) (Figure 1). Other phenolic compounds are also present in ginger, as quercetin, zingerone, gingerenone-A, and 6-dehydrogingerdione.

The maturation state, cultivar, environment, and processing steps are major factors that influence the biosynthesis and concentration of bioactive compounds in ginger. Besides, different composition of normal ginger and black ginger from different countries has been reported, evidencing that gingerol-related phenolic acids were present in normal ginger, while black ginger was characterized by the presence of methoxyflavones (26).

## Bioavailability and Pharmacokinetics

Ingested dietary gingerols need to be available in the circulation and tissues to produce an effect in the organism. Multiple factors influence the amount of a compound distributed to the different tissues to exert its action, including the solubility in the gastrointestinal fluid and possible degradation in gastrointestinal tract, permeability of enterocytes membrane, protein-mediated intestinal efflux or pre-systemic gut and/or hepatic metabolism (27).

Nutritional and clinical use of ginger in nutraceuticals or enriched-food products is limited due to its poor bioavailability. Gingerols and derivatives are lipid soluble compounds and therefore it would be expected a good absorption by passive diffusion across intestinal epithelium. However, prior to absorption, they must reach brush border cells, what implies be solubilized in an aqueous media; due to their chemical structure, they present a low solubility in water. This phenomenon is related to the concept Bioaccesibility, the amount of ingested nutrient available for absorption, which is different to the concept Bioavailability, that represents a step forward, that is, the portion of the ingested dose of a compound that reaches the general circulation and specific sites where it can exert its action. Bioaccesibility is the first limiting step in whether or not a compound may exert an effect in the organism (28).

Unlike other types of compounds, such as flavonoids, gingerols are not naturally present in glycosylated form, so that they are not hydrolyzed by glycosidase enzymes of intestinal brush border. However, gingerols are substrates of P-glycoprotein. This protein is highly expressed in the outer membranes of the enterocytes in the small intestine, as well as in liver, brain and kidney. It behaves as a major barrier to the intestinal absorption of many drugs, as a defense mechanism against toxics (29).

Once absorbed, gingerols are carried by the hepatic portal vein to the liver and undergo hepatic metabolism or “first-pass effect.” Half-life of these compounds is extremely low and they suffer from Phase II conjugative reactions, such as glucuronidation catalyzed by UDP-glucuronosyl-transferases (UGTs) and sulphation by sulphotransferases (SULTs), producing more polar molecules for biliary or renal excretion. Isoforms UGT1A1, 1A3 and 2B7 are responsible for gingerol conjugation (30).

**Figure 2:**
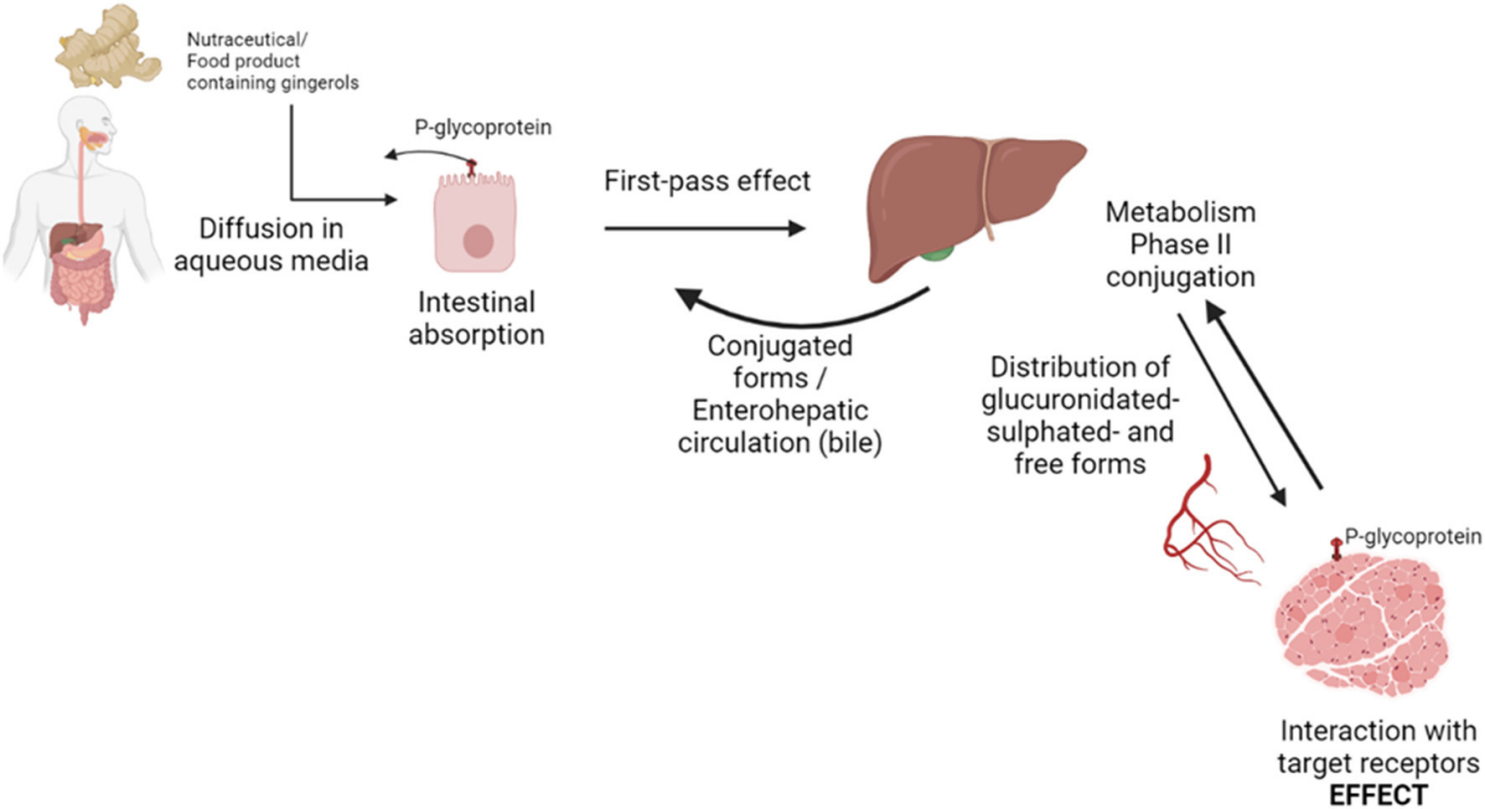
Scheme of bioaccessibility and bioavailability routes of gingerols in the organism.

Moreover, enterohepatic circulation occurs with these compounds, by biliary excretion and intestinal reabsorption. The hydrophilic metabolite 6-gingerol glucuronide diffuses out of the hepatic cells and is secreted in the bile into the small intestine. There it can be hydrolyzed by intestinal β-glucuronidases and re-enter into the blood stream through the enterocyte (31). All these phenomena are associated to extended half-life in plasma and prolonged pharmacological effect (Figure 2).

### Human Studies on Bioavailability of Gingerols

Most studies on ginger activity and bioavailability have been performed in animal studies and human trials are scarce. Zick et al. (32) investigated the pharmacokinetics of 6-, 8- and 10-gingerol and 6-shogaol and related metabolites in healthy subjects, with doses ranging from 100 mg to 2 g. The compounds showed a rapid absorption, as glucuronide metabolites appeared within 1 h and the elimination half-lives ranged between 75 and 120 min, depending on the administered dose. All detected compounds were glucuronide conjugates, and no free forms were detected.

The authors used an HPLC method with LOQ ranging from 0.1 to 0.25 μg/mL. The determination of bioactive phytochemicals and their metabolites presents the difficulty of the low concentrations at which they are found in biological fluids. The development of highly sensitive techniques such as mass spectrometry coupled to liquid chromatography has made it possible to better detect and quantify metabolites in animal and human studies after ingestion of ginger or food products made with ginger. The same authors developed and validated a more sensitive, LC-MS/MS method to characterize the pharmacokinetics of 6-, 8-, and 10-gingerols and 6-shogaol in human plasma and colon tissues (33). After an oral dose of 2 g of ginger extract (GE), concentrations of free 10-gingerol and 6-shogaol were detected (peak concentrations of 9.5 and 13.6 ng/mL, respectively). Most compounds existed as glucuronide and sulfate metabolites, mainly 6-gingerol-glucuronide (0.47 μg/mL). LOQ was established in 5 ng/mL, with similar T_max_ between 45 and 60 min and half-lives of all compounds and their metabolites between 1 and 3 h. Peak concentrations of sulfate metabolites were lower than glucuronide, being the higher value for 6-gingerol-sulfate (0.28 μg/mL). The multiple doses treatment consisted of 250 mg GE capsules daily for 28 days and no accumulation was observed for any of the quantified compounds, due to their short half-lives and fast clearance.

These studies have estimated the concentrations of gingerol glucuronides as the difference of gingerol concentrations prior to and after β-glucuronidase hydrolyzation, and not directly quantifying each compound. In this sense, Schoenknecht et al. (34) developed a direct liquid chromatography-tandem mass spectrometry method, using stable isotope synthesized standards of glucuronide forms, to detect and quantify gingerol glucuronides in human plasma. After SPE extraction and LC-MS analysis, the authors showed that the consumption of 1 liter of ginger tea led to a fast absorption and metabolization of gingerols, with maximum concentrations reached at 30 min post-ingestion. Plasma concentrations resembled the levels of each gingerol free form in the food product, with maximum plasma concentrations for 6-gingerol glucuronide (623.3 nmol/L), followed by 8-gingerol glucuronide (103.8 nmol/L) and 10-gingerol glucuronide (25.8 nmol/L). The authors collected pharmacokinetic parameters in plasma and urine, observing that the maximum concentrations and half-life in plasma were related to the carbon chain length and therefore to the hydrophobicity of the molecules. Pharmacokinetic parameters of urinary elimination indicated that the more lipid-soluble compounds remained longer in the body. 6-gingerol was still quantified in the interval 9–12 h. Recovery rates were between 45% of the administered dose for 6-gingerol and 10 % for 8-gingerol, expressed as glucuronide derivatives.

Some authors have hypothesized that glucuronide forms (inactive) interconvert to free (active) species in tissues by the presence of β-glucuronidase enzyme, establishing an equilibrium between both forms, what has been called “reverse pharmacokinetics.” The free form would exert its effects on its multiple target receptors. This might explain the disconnect observed between the efficacy of free gingerols and their sub-therapeutic plasma concentrations (35). The authors demonstrated the accumulation of conjugated forms within various tissues, including brain, after repeated daily oral administration of ginger extract at 250 mg/kg for seven days.

### New Technologies to Improve the Bioavailability of Ginger Bioactive Compounds

Low bioavailability of gingerols has been related to its poor water solubility and excessive phase II hepatic metabolism. Different strategies have been implemented to enhance the bioavailability of poorly water-soluble compounds. These technologies include nanoparticles, micelles, emulsions or solid dispersion (36, 37), liposomes (38) or self-microemulsifying drug delivery systems (39). Studies performed in animal models on these forms of encapsulation of single and combined ginger compounds revealed better pharmacokinetic profiles in all cases.

Xu et al. (39) conducted a bioavailability study with a 6-gingerol-loaded self-microemulsifying drug delivery system (SMEDDS) for oral administration in rats. It was formulated with 250 mg/kg dose of 6-gingerol and the system consisted of a mixture of oil phase and surfactants, creating an oil-in-water microemulsion. The 6-gingerol-SMEDDS exhibited prolonged plasma circulation, and significant higher absorption than free 6-gingerol (t_1/2_ = 210 min and AUC = 2,987 min μg/mL, compared to free form t_1/2_ = 82 min and AUC = 454 min μg/mL).

Similar results were observed by Wei et al. (40), who developed nanostructured lipid carriers (NLC) to improve oral solubility and bioavailability of 6-gingerol. After oral administration in rats, AUC was significantly higher compared to controls. Encapsulation of the drug in a lipid core coated with surfactants might help to first, increase the diffusion to epithelial space and improve the absorption and second, to avoid the first-pass effect. The small particle size contributes to a greater surface/volume ratio and major absorption.

Liposomes are new drug carriers prepared by the formation of vesicle enveloping drug molecules in the phospholipid bilayer membrane. Wang et al. (38) demonstrated that 6-gingerol encapsulated in proliposomes was retained in the blood stream much longer than the free form. The plasma concentration was significantly higher 30 min after oral administration of a dose of 250 mg/kg in rats.

Another approach conducted by Ogino et al. (31) was the solid dispersion of ginger extract (GE). Solid dispersions consist of a dispersion of a drug in a solid matrix made of either a small molecule or a polymer. The dispersed drug can exist in different isoforms or crystallization states. In this study, the solid dispersion was made using a hydrophilic polymer, hydroxypropyl cellulose, by a freeze-drying technique. Oral absorption (dose administered 100 mg GE/kg) was higher than that of GE alone, with enhanced AUC and C_max_ of each gingerol. 6-gingerol and 8-gingerol showed 5-fold higher bioavailability than their respective counterparts in free GE.

In an acute study with doses of 250 mg/kg of 6-gingerol administered in rats, polyethylene glycol-based polymeric micelles significantly improved (up to 3-fold) the bioavailability of 6-gingerol compared to 6-gingerol control group (37). Besides, there was a better brain distribution, what suggested that the micelle could overcome the brain-blood barrier. It has been hypothesized that the components of the micelles work as P-glycoprotein inhibitors, by suppressing its ATPase activity, hence improving the passage through biological barriers (41, 42).

In all studies, *in vitro* release assays were performed and an enhanced solubility of the compound was observed, compared to the free drug, which could be partly responsible of the improved oral bioavailability in circulation. Nevertheless, further research is required to confirm the usefulness of these preparations as nanocarriers, as well as thorough toxicity studies prior to human administration.

## Antioxidant and Inflammatory Activity

The production of free radicals, such as reactive oxygen species (ROS) or nitrogen reactive species (NOS) leads to the development of many oxidative-related disorders, such as the most common neurogenerative diseases (43). Hopefully, antioxidant bioactive compounds are widely spread over a large number of food matrices as fruits, vegetables, cereal grains, edible flowers or medicinal plants (44–50). Moreover, the latest scientific literature has found promising bioactive compounds contained in ginger that possess antioxidant and anti-inflammatory activities (20, 51, 52). Gingerols and shogaols have a plethora of biological activities such as antioxidant, antimicrobial, anticancer, anti-inflammatory, antiallergic and prevention of neurodegenerative diseases (7).

The antioxidant activity of ginger has been evaluated *in vitro*, showing better performance for dried ginger compared with fresh, stir-fried or carbonized ginger. This fact was principally related with the concentration of polyphenols, higher in dried ginger, as the temperatures applied for stir-fried or carbonized ginger could change gingerols into shogaols, leading to minor antioxidant capacity (53).

Moreover, the scientific literature has reported that ginger can be useful for the prevention of oxidative-related injury (51, 54). An extract from ginger showed antioxidant capacity related to interleukin-1β in human chondrocyte cell model, stimulating the expression of enzymes related to oxidative protection, reducing the generation of ROS leading to decreased lipid peroxidation (55). Ginger extract was also able to reduce ROS in human fibrosarcoma cells (56). Besides, another marker of lipid peroxidation as malondialdehyde was reduced in rat heart homogenates after the treatment with a ginger extract (54).

Particularly, ginger has shown antioxidant capacity *via* the nuclear factor erythroid 2-related factor 2 signaling pathway (Nrf2) (57, 58). In human colon cancer cells 6-shogaol is able to increase intracellular glutathione/glutathione disulfide ratio (GSH/GSSG), upregulating the expression of Nrf2, metallothionein 1 (MT1), heme oxygenase-1 (HO-1), ferritin light chain (FTL), aldo-keto reductase family 1 member B10 (AKR1B10), and γ-glutamyltransferase-like 4 activities (GGTLA4).

Despite the fact that doses, routes of administration and duration of treatment vary among studies, it has been reported that the effective anti-inflammatory and antioxidant doses of ginger extract *in vivo* studies range from 200 to 500 mg/kg/day, and the effective immunomodulatory doses range from 28 to 720 mg/kg/day. In human studies doses of 500 mg/day for 3 months, 1,000 mg/day for 2 months and 1,500 mg/day for 6 weeks were observed (20). Doses of up to 4 grams of ginger per day have been reported to be safe (59).

**Figure 3:**
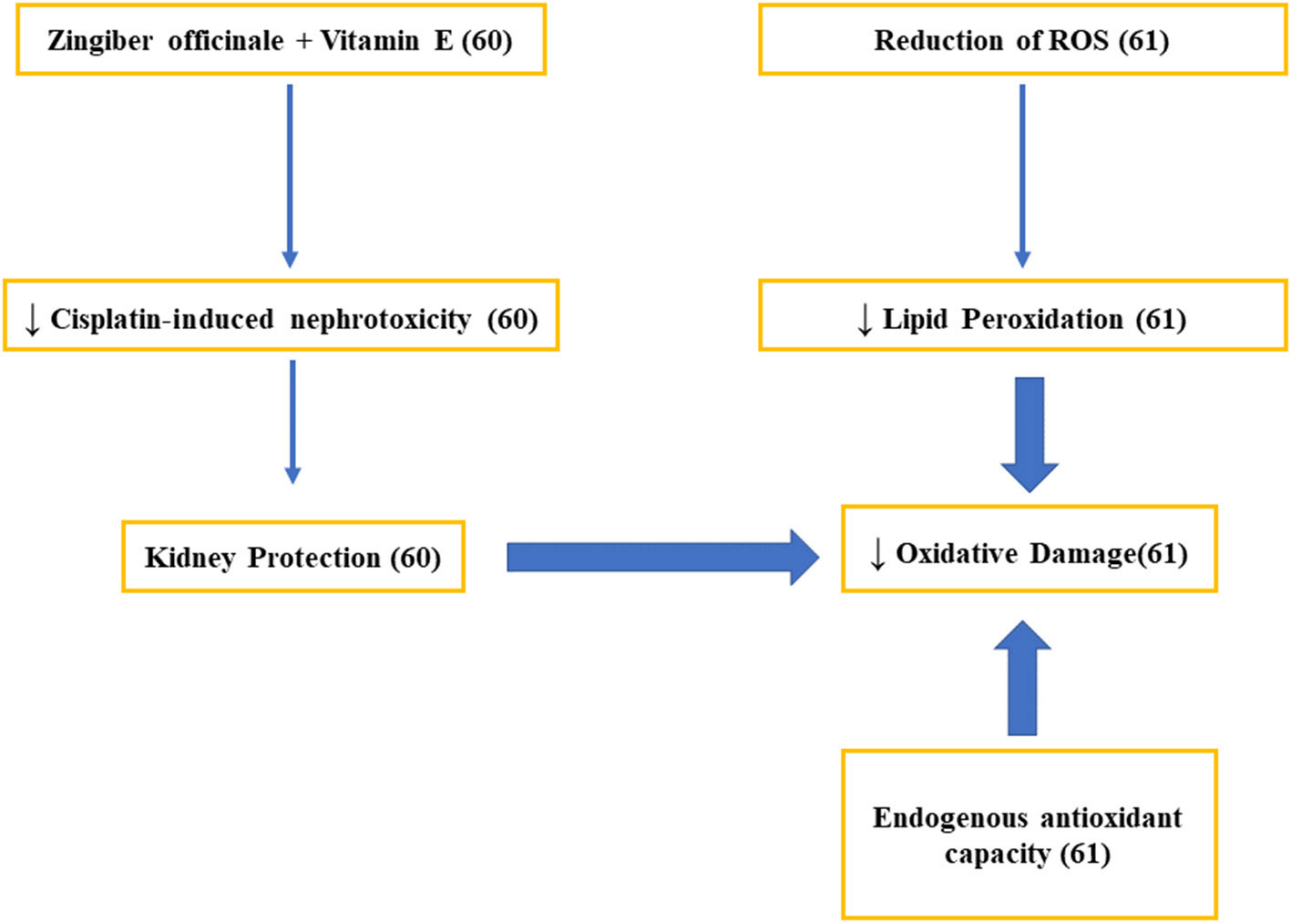
Studies on the mechanisms related to the antioxidant activity of ginger.

Both *in vitro* and *in vivo* studies have revealed that ginger and related bioactive secondary compounds, such as 6-shogaol, 6-gingerol, and oleoresin, exert potent antioxidant capacity by direct free radical scavenging. Additionally, the triggering of the Nrf2 signaling route is decisive to the underlying mechanisms of action. Importantly, the excess on the production of ROS and NOS is considered a cause of some diseases as neurodegenerative pathologies and antioxidants are crucial for their prevention (60, 61) (Figure 3).

**Figure 4:**
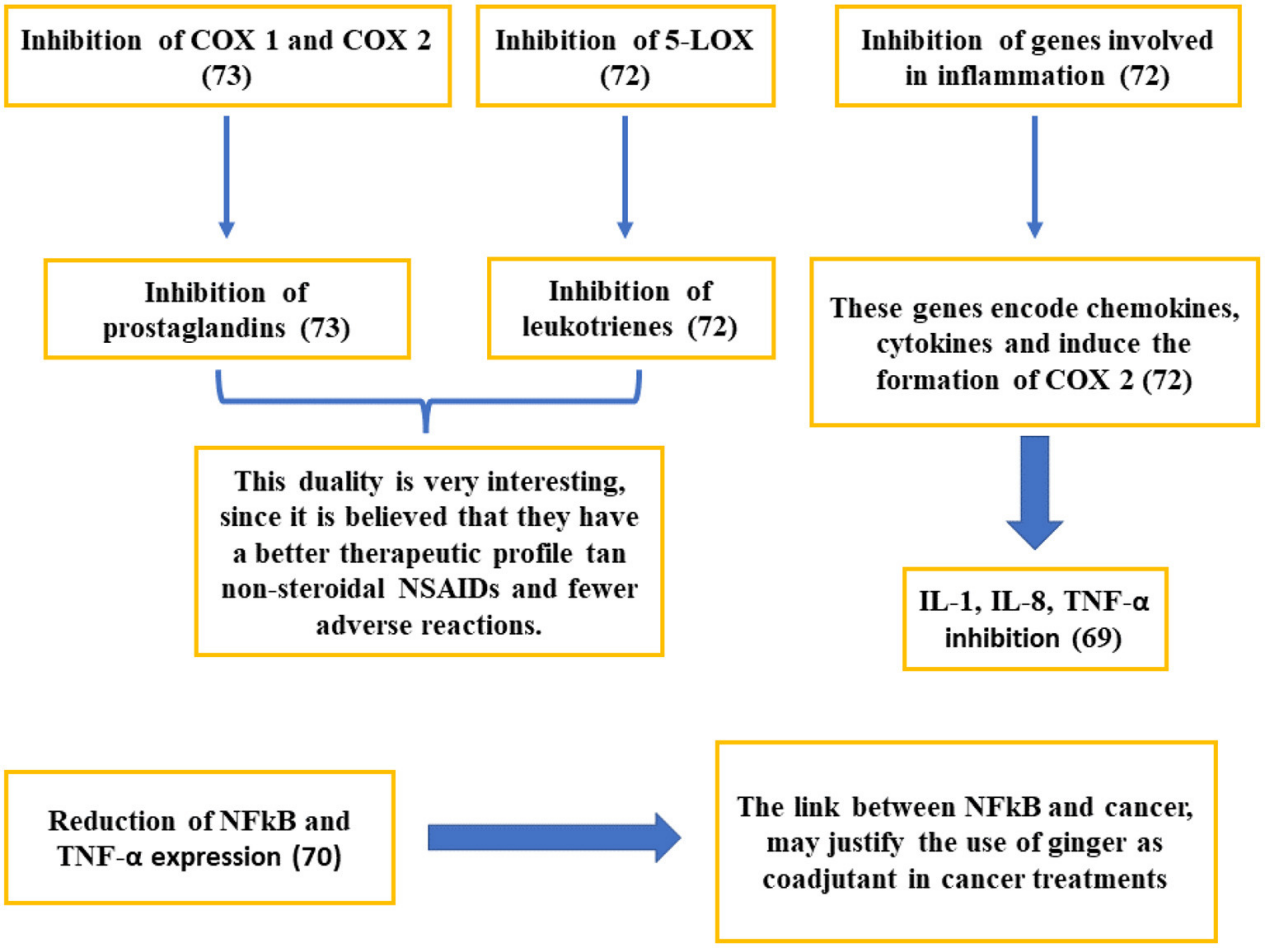
Studies on the mechanisms of action related to the anti-inflammatory activity of ginger.

Results on the anti-inflammatory capacity of ginger and its bioactive compounds have shown some variability (62, 63), which may be attributed to differences in the study design, length of interventions, individual characteristics, and doses administered. The anti-inflammatory mechanisms of ginger are probably associated with a decline in proinflammatory cytokines linked to the inhibition of Akt and NF-κB activation (8). NF- kβ pathway is widely used by eukaryotic cells as a regulator of genes that control cell proliferation and cell survival. NF-kβ is the key regulator of the inflammatory process, activating the expression of inflammatory target genes, including cytokines, chemokines, and COX2.This enzyme triggers the formation of some prostaglandins, responding to inflammation and enhancing the formation of proinflammatory cytokines. Ginger has being able to inhibit inflammatory response by suppressing NF-kβ, which lead to the reduction of cytokine gene expression (11). In 2016, a meta-analysis reported that C-reactive protein (CRP) and other acute-phase proteins were also suppressed after ginger supplementation (64). Naderi et al. (65) published that treatment for 12 weeks with ginger powder at a dose of 1 g/day was able to decrease the plasma concentration of CRP, in accordance to previous studies (66). Likewise, the anti-inflammatory capacity of ginger can be justified by its ability to inhibit COX-2 and 5-lipoxygenase enzymes, which results in the suppression of amino acid metabolism. In fact, it has demonstrated to reduce platelet aggregation, as well as the formation of pro-inflammatory thromboxanes and prostaglandins (67). Specifically, the anti-inflammatory effects of ginger are related to the inhibition of COX-2 without affecting COX-1, which seems to be an advantage over traditional NSAIDs due to the related side effects (68, 69). Van Breemen et al. through pulsed ultrafiltration mass spectrometry, showed that several compounds related to gingerol were COX-2 ligands. COX-2 inhibition would prevent the conversion of arachidonic acid into prostaglandin (PG) H2, preventing its subsequent conversion into proinflammatory prostaglandins such as PGD2 and PGE2 (68). It has also been reported the inhibition of the formation of nitric oxide, inflammatory cytokines, and the inhibition of the enzymatic activity of prostaglandin synthase, which could lead to a decrease in the inflammatory component (69–73) (Figure 4).

Health state, genetics, lifestyle habits and dietary factors of individuals, or the dosage and solubility aspects of ginger forms could affect the bioaccessibility and bioavailability and ultimately the bioactivity of ginger compounds, which may justify the contradictory or controversial results emerged from *in vitro* and *in vivo* studies.

## Alzheimer's Disease and Ginger

Alzheimer's disease (AD) is a neurodegenerative condition linked to profound memory impairment and loss of cognitive function. Among others, cellular damage due to β-amyloid protein aggregation, tau protein hyperphosphorylation, neurotransmitter imbalances, oxidative stress, apoptosis and inflammatory responses is responsible for its occurrence (3, 74).

Due to the inadequate efficacy of the conventional drugs currently used, their adverse effects and pharmacokinetic problems, together with the scientific evidence that in recent years suggests that traditional medicinal plants could be useful both in the prevention and treatment of a multitude of pathologies, a great opportunity has led for their evaluation in the treatment of memory disorders, as it is the case of *Zingiber officinale* (3, 75).

The main characteristics of *Zingiber officinale* for its possible use in neurodegenerative diseases, specifically Alzheimer's, are its anti-inflammatory and antioxidant effects. In particular, clinical studies have shown that the use of ginger has increased the expression of nerve growth factor (NGF), playing a key role in improving memory function, simplifying long-term hippocampal enhancement and accelerating neurite outgrowth.

Preclinical trials in mice (Table 1) showed that increasing NGF levels in the hippocampus initiated the activation of extracellular signal regulatory kinases (ERK) and cAMP response element binding protein (CREB), leading to increased synaptogenesis (76). Furthermore, studies have shown that ginger blocks the expression of pro-inflammatory cytokines and chemokines in THP-1 cells. Animal studies concluded that the use of ginger significantly inhibited the expression of mRNA related to the expression of pro-inflammatory cytokines and endothelial adhesion activating factors such as LPS, TNF-α, IL-1 β, COX-2, MIP-1A, MCP-1 and IP-10, among others (77).

**Table 1:** Preclinical studies of ginger and Alzheimer's disease.

**Model Animal**	**Compound used**	**Conclusions**	**References**
Mouse	Ginger	AChE activity, cytotoxic Effect, Lps-induced NO production, Supressed TNF-a, IL-6, IL-1B	(78)
Rat	6-gingerol	IL-6, TNF-α, NO, NOS_2_ protein expresión in C6 cells.	(15)
Male Swiss albino mice	Gingerol	Reduction of the levels of AB42, B-secretase, APH1a and COX-2. Increased of a-secretase activity	(79)
Male Wistar rats	Extract of *Cyperus rotundus* and *Zingiber officinale*	Oxidate stress reduction in hippocampus improved cholinergic gunction, improved memory deficit	(80)
Male C57BL/6 mice	Ginger, 6-Gingerol	Upregulation of BDNF. Prevention of memory deficits	(81)

*In vitro* and animal studies conclude that various bioactive compounds of *Zingiber officinale* cross the blood-brain barrier, allowing us to think that the beneficial properties observed in diverse pathologies could have application against neurodegenerative diseases, specifically AD (82).

*Zingiber officinale* might also have therapeutic properties for other diseases affecting the nervous system, such as brain tumors, cardiovascular accidents, neurosis, depression, insomnia and psychiatric disorders. It is included on the US Food and Drug Administration's (FDA) “Generally Recognized as Safe” (GRAS) list and can be defined as a safe nutraceutical that could be used to combat neurodegenerative disorders (75).

However, clinical studies in humans are scarce and some of them refer to supplements consisting of a mixture of herbs, including ginger, used in traditional oriental medicine, as Davaie Loban or Kihito (Table 2). Other authors have reported improvements in cognitive abilities using Cognitex, a nutritional supplement containing sage, blueberry and Zingiber officinale. Saenhong et al. (83) evaluated the individual effect of ginger and findings are noteworthy. The researchers conducted a placebo-controlled study with standardized ginger extracts, observing an enhance in cognitive processing capabilities, with greater effects at higher doses of 800 mg/day (Table 2).

**Table 2:** Human clinical studies on ginger and cognitive function.

**Study design**	**Intervention**	**Outcomes**	**References**
Elderly people with memory complaints. Open label study (*n* = 30)	“Cognitex” (12 weeks)	↑ memory abilities ↑ sustained attention ↑ visual learning ↑ activities of daily living	(84)
Double-blind randomized placebo-controlled. Middle-aged healthy Thai women (*n* = 60)	Placebo, 400, 800 mg/day standardized ginger extract (8 weeks)	↑ working memory ↓ latency to audit stimuli ↑ Word recognition ↑ Choice reaction time ↑ Numeric working memory ↑ Spatial working memory	(83)
Double-blind randomized placebo-controlled > 50 years, mild to moderate AD patients (*n* = 50)	Placebo, Davaie Loban capsules 500 mg, 3 times day (12 weeks)	Improvements in AD scores (ADAS-cog, CDR-SOB)	(85)
Open-label, crossover AD patients (*n* = 10)	Kihito herbs 2,5 g extract, 3times/day (16 weeks)	Improvements in AD scores (MMSE-J)	(86)

*ADAS-cog, Alzheimer's Disease Assessment Scale-cognitive subscale; CDR-SOB, Clinical Dementia Rating Scale Sum of Boxes; MMSE-J, Mini-Mental State Examination-Japanese*.

## Parkinson Disease and Ginger

Parkinson's disease (PD) is a complex neurodegenerative process that appears in adulthood and is the second most common neurodegenerative disease behind Alzheimer's dementia. Its pathological basis is characterized by the progressive loss of dopaminergic neurons of the substantia nigra pars compacta (SNpc) of the midbrain, as well as the presence of intracellular inclusions called Lewy bodies, which are formed by insoluble aggregates of abnormally folded alpha-synuclein protein. The result of this neurodegeneration is the dopaminergic denervation of the projections of the SNpc toward the striatum, which conditions an alteration in the normal physiology of the basal ganglia (87, 88). These phenomena leads to a deficit of dopamine (DA) and the subsequent appearance of the cardinal signs of the disease, that is, the tremor resting, bradykinesia, posture rigidity and instability. In addition to the motor symptoms, there is the manifestation of non-motor symptoms, the prevalence of which increases as the disease progresses (apathy or depression, sleep disturbances, autonomic dysfunction or sensory symptoms) (87, 89).

PD can be caused by hereditary and environmental factors, including oxidative stress and iron accumulation in the brain. It is clear that neuroinflammation plays an important role in the development and progression of PD and other neurodegenerative diseases (19, 90). In PD, oxidative stress is a result of mitochondrial deficiency, in addition to a chronic inflammatory process, in which both produce reactive oxygen species (ROS) and reactive nitrogen species (RNS). These reactive species meet the accumulated iron in the brain and harm structures, leading to the death of dopaminergic neurons in the substantia nigra. This process creates a cycle of cell damage, neuroinflammation, and ROS/RNS production, resulting in neuronal death (18, 91). The combination of oxidative stress and high levels of tissular iron cause harm to the brain structure, with the death of dopaminergic neurons in the substantia nigra. Consequently, the loss of these dopaminergic neurons in the substantia nigra lead to progressive motor impairment in PD (18). In fact, oxidative stress levels and inflammatory markers are significantly increased in PD patients.

Currently available treatments have a strictly symptomatic effect. The most effective drug for treating the motor manifestations of PD is levodopa. To date, there is no treatment that slows the progression of the disease, as current drugs improve the symptoms of PD but not the underlying neurodegeneration of PD. In recent years, interest has increased in the discover of the possible beneficial effect of natural products as ginger on the development and progression of PD. Park et al. (92) reported a neuroprotective effect of 6-shogaol in a PD model; 6-shogaol protected dopaminergic cells against MPP+ - and MPTP-induced neurotoxicity *via* the inhibition of neuroinflammatory responses of microglia. In this way, results of Moon et al. (76) suggest that 6-shogaol may play a role in inhibiting glial cell activation and reducing memory impairment in animal models of dementia (Table 3).

**Table 3:** Parkinson's disease and ginger.

**Effect**	**References**
Suppressing the overactivation of astrocytes Attenuated LPS-induced neuronal cell loss by reducing the expression of GFAP and IL-1ß in the hippocampus.	(90)
Low levels of antioxidants, incapable of controlling free radical and ROS/RNS production with subsequent inflammation, leading to neurodegeneration in PD.	(18)
Inhibited components of the inflammatory pathway such as TNF-α, NO, COX-2, and inducible nitric oxide synthase (iNOS)	(92)
6-shogaol may play a role in inhibiting glial cell activation and reducing memory impairment in animal models of dementia.	(76)

Kongsui et al. (90) suggested that ginger crude extract might be a potential neuroprotective agent for the treatment of lipopolysaccharide-induced neurodegenerative diseases. Other study carry out by Hussein et al. (93) clearly indicates a neuroprotective effect of ginger against MSG-induced neurodegenerative disorders and these beneficial effects could be attributed to the polyphenolic compounds present (Table 3).

## Multiple Sclerosis and Ginger

Multiple sclerosis (MS) is a chronic autoimmune disease of the central nervous system (CNS) characterized by inflammation, demyelination of neurons and axonal degeneration even in the early stages of the disease. MS is one of the most common causes of neurological disability in young people (94, 95) and usually appears in women with ages comprised between 25 and 30 years (95, 96).

Currently MS is considered as multifocal chronic inflammatory disease that associates neurodegeneration (97). Some individuals are genetically predisposed to such an abnormal autoimmune response, and the development and progression of the disease will be affected by various environmental factors. Genetic predisposition is mediated especially by the major histocompatibility complex. Among the risk factors with the best available evidence are the association with Epstein-Barr virus infection, high BMI during adolescence, low vitamin D levels and smoking (95, 96). The number of population affected by MS has increased in recent decades and it is estimated that 2.5 million people worldwide suffer from the disease, affecting some 700,000 population in Europe (96, 98).

As for the pathogenesis, despite decades of research, the exact etiology is still unknown to the scientific community and it is believed that the symptoms of MS result from damage to the myelin sheath and disruption of myelinated tracts in the CNS (99). In most patients, the characteristic clinical symptoms of the disease include cognitive, sensory, motor, and autonomic disturbances. These symptoms manifest as loss of coordination and balance, impaired vision, deficits in executive functioning, chronic pain and mood disturbance (94). There is currently no definitive cure for MS. However, different pharmaceutical and rehabilitation therapies are available to treat acute attacks, improve symptoms and modify the course of the disease (100). In recent years, complementary and alternative medicine methods such as the use of herbal therapy appear to have promising therapeutic approach to treat MS (101). Such therapies among which ginger is included, could be effective in the treatment of MS by reducing demyelination, enhancing remyelination and especially by suppressing/reducing inflammatory processes. Regarding the reduction of inflammatory processes, it occurs by inhibiting the infiltration of inflammatory cells in the CNS, reducing the proinflammatory cytokine production.

Demyelination and neurodegeneration are closely related to inflammation (a key feature in MS), being much more pronounced in acute and relapsing phases (101). Within the CNS there is an infiltration of leukocytes including neutrophils, DCs, macrophages, CD4+ T cells, and CD8+ T cells), with CD4^+^ T cells having the greatest impact on demyelination of neurons and axonal damage (101, 102). As for DCs they cross the damaged blood-brain barrier promoting a polarization of myelin-specific T-lymphocytes to different subsets of effector T-cells; Th1, Th2, Th9, Th17, Th22 and Treg cells. While Th1 and Th17 cells act pathogenically in the immunopathological process of MS, Treg and Th2 cells exert protective action against autoimmune diseases (103–105). Astrocytes and microglia cells also contribute to the pathogenesis of MS by releasing proinflammatory cytokines (20).

There are currently more than a dozen drugs on the market to treat MS. However, they are questioned both for their moderate efficacy and side effects. The possibility of using ginger to attenuate the symptoms of MS arises from the fact that there are certain components derived from plants with anti-inflammatory and immunomodulatory properties and with low side effects (20).

Among the possible therapeutic potentials of ginger and its components for the treatment of MS, its immunomodulatory, anti-inflammatory and antioxidant effects are depicted in Table 4, and their mechanisms are extensively detailed by Jafarzadeh et al. (20).

**Table 4:** The anti-inflammatory, antioxidant activities and immunomodulatory effects of ginger and its components.

	Th1 cell-related immune responses
	Th17 cell-related immune responses
	B cell-related immune responses
**Down-regulation**	Antigen presenting cells
**of the**	Arachidonic acid-derived mediators
	Oxidative stress Expression of the chemokines and chemokines receptors
	Adhesion molecules
	Th2 cell-related immune responses
	Th9 cell-related immune responses
	Th22 cell-related immune responses
**Modulation of the**	Macrophage's responses
	Production of pro- and anti-inflammatory cytokines
	Toll-like receptor's-related signaling pathways
	Inflammasome-related responses
**Up-regulate of the**	Treg cell-related immune responses4

According to a recent systematic review on the concomitant consumption of ginger extract and other drugs it can be concluded that ginger consumption is safe and there is no potential risk of clinically relevant interactions in the treatment of MS (106). The only contraindications were observed in the coadministration together with anticoagulants, due to the anticoagulant properties of ginger.

Experimental autoimmune encephalomyelitis (EAE) is a model of inducible human MS in vulnerable animals. It is usually induced in mice due to the fact that it is a highly reliable model to study both the pathogenesis of MS to test drugs in development to treat MS (107, 108). In different studies performed in mice with EAE, it was observed that after the administration of ginger extract the clinical symptoms of EAE appeared later and the clinical scores of the disease were lower compared to placebo (109, 110). The main feature of MS and EAE is primary demyelination of axons, causing blocking of signal conduction or reduced conduction at the demyelinated site (111). Administration of ginger extract prior to EAE appears to reduce the clinical symptoms, through up-regulation of inflammatory cytokines and chemokines (IL-23, IL-33, IFN-γ, CCL20 and CCL22) (109, 112). Moreover, a recent investigation in mice with EAE showed that both 6-shogaol and 6-paradol appear to reduce clinical symptoms. In addition, they were also associated with attenuation of astrogliosis, microglial activation and TNF-α expression, suppressing neuroinflammatory responses. Therefore, it seems that 6-shogaol and 6-paradol could be the active ingredients responsible for the efficacy of ginger extract (111).

The research in mice with EAE seems to be effective to further our knowledge of all the possible mechanisms involved in the pathogenesis and treatment of MS, due to the similarities (113). Therefore, although more research is needed, we consider it a promising and necessary first step for the assessment of the efficacy prior to human studies.

## Conclusions

Ginger contains diverse bioactive compounds, such as gingerols, shogaols, and paradols and possesses antioxidant and anti-inflammatory properties that might help reduce the levels of inflammation and oxidative stress in neurodegenerative diseases. In fact, several inflammatory, oxidative and immunopathological parameters are involved in their pathogenesis and drugs used for treatment are of limited efficacy and can also generate adverse side effects. It seems that ginger, given its antioxidant, immunomodulatory and anti-inflammatory capacity, has the ability to intercept all the main elements involved in the development of multiple sclerosis as well as to attenuate the symptoms of neurological diseases including Parkinson's, Alzheimer's, migraine, and epilepsy. Even though with the doses studied, no considerable adverse effects are observed, further research is needed to study whether higher doses and/or longer administration protocols are more effective without causing adverse side effects.

Inclusion of ginger or ginger extracts in nutraceutical formulations could provide valuable protection against neurodegenerative diseases. The low bioavailability and extensive phase II metabolism have limited the use of ginger in neurodegenerative pathologies and new pharmaceutical forms for delivering ginger's bioactive compounds that overcome these limitations are currently being developed. Further toxicological and pharmacokinetic studies of these new formulations will be necessary before their application in human trials, but the evidence is promising for the therapeutic potential of ginger in neurodegenerative diseases.

## Author Contributions

PZ: conceptualization, supervision, and project administration. JM, BC, RA, MC, PZ, and DV: methodology, investigation, and writing—original draft preparation. JM, BC, RA, PZ, and DV: writing—review and editing. All authors have read and agreed to the published version of the manuscript.

## Conflict of Interest

The authors declare that the research was conducted in the absence of any commercial or financial relationships that could be construed as a potential conflict of interest.

## Publisher's Note

All claims expressed in this article are solely those of the authors and do not necessarily represent those of their affiliated organizations, or those of the publisher, the editors and the reviewers. Any product that may be evaluated in this article, or claim that may be made by its manufacturer, is not guaranteed or endorsed by the publisher.
